# Fibroblast growth factor 23 is associated with proteinuria and smoking in chronic kidney disease: An analysis of the MASTERPLAN cohort

**DOI:** 10.1186/1471-2369-13-20

**Published:** 2012-04-24

**Authors:** Marc G Vervloet, Arjan D van Zuilen, Annemieke C Heijboer, Piet M ter Wee, Michiel L Bots, Peter J Blankestijn, Jack FM Wetzels

**Affiliations:** 1 Department of Nephrology and ICaR-VU, VU university medical centre, Amsterdam, The Netherlands; 2 Department of Nephrology, UMCU, Utrecht, The Netherlands; 3 Department of Clinical Chemistry, VU university medical centre, Amsterdam, The Netherlands; 4 Julius Center for Health Sciences and Primary Care, University Medical Center Utrecht, Utrecht, The Netherlands; 5 Department of Nephrology, Radboud University Nijmegen Medical Center, Nijmegen, The Netherlands

**Keywords:** Cardiovascular disease, CKD, FGF23, Phosphate, Proteinuria, Smoking

## Abstract

**Background:**

Fibroblast growth factor 23 (FGF23) has emerged as a risk factor for cardiovascular disease and mortality throughout all stages of chronic kidney disease (CKD), independent from established risk factors and markers of mineral homeostasis. The relation of FGF23 with other renal and non-renal cardiovascular risk factors is not well established.

**Methods:**

Using stored samples, plasma FGF23 was determined in 604 patients with moderate to severe kidney disease that participated in the MASTERPLAN study (ISRCTN73187232). The association of FGF23 with demographic and clinical parameters was evaluated using multivariable regression models.

**Results:**

Mean age in the study population was 60 years and eGFR was 37 (± 14) ml/min/1.73 m^2^. Median proteinuria was 0.3 g/24 hours [IQR 0.1-0.9]. FGF23 level was 116 RU/ml [67-203] median and IQR. Using multivariable analysis the natural logarithm of FGF23 was positively associated with history of cardiovascular disease (B = 0.224 RU/ml; p = 0.002), presence of diabetes (B = 0.159 RU/ml; p = 0.035), smoking (B = 0.313 RU/ml; p < 0.001), phosphate level (B = 0.297 per mmol/l; p = 0.0024), lnPTH (B = 0.244 per pmol/l; p < 0.001) and proteinuria (B = 0.064 per gram/24 hrs; p = 0.002) and negatively associated with eGFR (B = -0.022 per ml/min/1.73 m^2^; p < 0.001).

**Conclusions:**

Our study demonstrates that in patients with CKD, FGF23 is related to proteinuria and smoking. We confirm the relation between FGF23 and other cardiovascular risk factors.

## Background

Chronic kidney disease (CKD) is a well-established risk factor for cardiovascular disease and mortality [1]. The added risk of CKD is both graded, depending on stage of disease, as well as independent from traditional cardiovascular risk factors. Many studies, both experimental and epidemiological, have tried to define the components typical for CKD that carry this added risk. Proteinuria [2], malnutrition, deranged homeostasis of calcium and phosphate, vitamin D deficiency, anemia, and hyperparathyroidism have all been implicated to be involved. Recently, fibroblast growth factor 23 (FGF23) was identified as a novel risk factor not only in patients with end stage kidney disease [3] and advanced stages of CKD [4,5], but also in patients in the early stages of CKD and in the general population [6]. Since FGF23 has an important role in the regulation of mineral balance and vitamin D metabolism, it is intriguing that its contribution to the increased risk was independent from calcium, phosphate and vitamin D. These observations supported the hypothesis that FGF23 may exert biological and possibly pathological effects that are not mediated through calcium, phosphate or vitamin D. Theoretically FGF23 could have direct toxic effects or influence non-mineral cardiovascular risk factors. Indeed it was suggested that FGF23 influenced vascular function in humans [7] and was associated with heart function and atherosclerosis [8]. Most recently, strong evidence suggested a direct role for FGF23 as an inducer of left ventricular hypertrophy in a mouse model [9].

In the current study we investigated the association of FGF23 with established cardiovascular risk factors, and with additional specific renal risk factors for cardiovascular disease in a large well-described cohort of patients with moderate to severe CKD. The mutual relationship between FGF23 and these established risk factors could provide important clues for future therapy, aiming at reducing cardiovascular risk.

## Methods

### Patients

For the current study patients from the MASTERPLAN study were used [10]. The MASTERPLAN study was centrally approved by the medical ethical committee of UMCU (METC, University medical center Utrecht), and locally by all participating centers. Criteria for patient eligibility and methods of data collection of MASTERPLAN have been described previously. In short, MASTERPLAN is a randomized controlled clinical trial (ISRCTN73187232), performed in nine Dutch hospitals. 788 patients with CKD (eGFR 20-70 ml/min/1.73 m2) were randomized to either receive usual care by the nephrologist or intensified treatment with added nurse practitioner support. All participants gave written informed consent. The primary end point is a composite of fatal and nonfatal myocardial infarction, stroke and cardiovascular mortality. Inclusion started in 2004 and ended in December 2005.

For the current analysis we retrieved blood samples collected at baseline by the patients that participated in the MASTERPLAN study. We excluded patients who had received a kidney transplant (n = 184).

### Data collection

Baseline measurements included a questionnaire recording smoking behavior, physical activity and medication use. Physical examination consisted of measurement of height, weight and blood pressure. Blood was drawn and a 24-hour urine sample was collected. Blood and urine samples were analyzed by the laboratory of the local center. Medical history was obtained from the medical records. History of CV disease was defined as a history of myocardial infarction, ischemic stroke or vascular intervention. Diabetes mellitus was defined as the use of glucose lowering drugs or a fasting glucose > 126 mg/dl (7.0 mmol/l).

### FGF23 and vitamin D measurements

EDTA-plasma has been stored at -80°C until use. In these samples c-terminal FGF23 was determined in duplicate using a sandwich ELISA (Immutopics, San Clemente CA) with an intra-assay and interassay coefficient of variation of < 5% and < 16% respectively [11]. Glomerular filtration rate was estimated (eGFR) with the four-point MDRD formula [12]. Proteinuria was determined in 24-hour urine collections.

In selected samples that were used for the FGF23 assay, both 25vitD and 1,25vitD were determined using a RIA (Diasorin, Stillwater, MN and IDS, Tyne and Wear, United Kingdom, respectively). Intra-assay and interassay coefficient of variation were < 7% and < 10% for D25 and < 9% and < 11% for 1,25D respectively.

### Statistical analysis

Using multivariable analysis the relation of FGF23 with classical risk factors for cardiovascular disease was studied (model 1, sex, age, blood pressure, presence of diabetes, prior cardiovascular disease, baseline body mass index (BMI) and total cholesterol levels). Next, in the fully adjusted model, the so-called "renal risk factors" were added to the model, and the use of active vitamin D (model 2: model 1 plus calcium, phosphate, PTH, eGFR, proteinuria, and use of active vitamin D). To study a possible graded relation between proteinuria and FGF23, all patients were divided, according to the degree of proteinuria: 1) no proteinuria; 2) proteinuria < 0.5 gram/24 hours; 3) proteinuria between 0.5 and 2.0 gram/24 hours and 4) proteinuria > 2 gram/24 hours. For skewed data (like FGF23) log-transformation was performed. As we found during analysis an association between proteinuria and FGF23, we subsequently determined serum level of 25vitD and 1,25vitD in all patients with proteinuria > 2 gram/24 hours (n = 63) and in a random sample of equal size of patients with proteinuria < 2 gram/24 hours, to study the possible confounding effects of vitamin D levels on this relation. The threshold of 2 gram/24 hours was arbitrarily chosen to have a sufficient number of patients above this level. In this subgroup multivariable analysis was performed with FGF23 as dependent variable, and levels of vitamin D were added to the fully adjusted model 2. A dichotomous variable was subsequently used (presence or absence of proteinuria above 2 grams/24 hours) to account for the categorical effect of high-proteinuria on FGF23 levels.

## Results

### Patients

For 604 patients at baseline a plasma sample was available for determination of FGF23, and all these patients were included. Mean age was 60.2 (sd 12.5) years, 69% was male and mean eGFR was 37 (sd 14) ml/min/1.73 m^2^. All other baseline characteristics are shown in Table 1.

**Table 1:** Baseline characteristics

Variable	N(%) = 604(100%)
**Gender (male)**	417(69%)
**Age (yrs)**	60.2(± 12.5)
**BMI (kg/m^2^)**	26.5(24.3-29.4)
**Race (Caucasian)**	560(92.7)
**Cause of CKD**	
**Diabetic nephropathy**	65(10.8%)
**Renovascular**	188(31.1%)
**Glomerular disease**	109(18%)
**Interstitial disease**	57(9.4%)
**Congenital (incl PKD)**	62(10.3%)
**Unknown**	123(20.4%)
**History of CVD**	178(29.5%)
**Diabetes Mellitus**	144(23.8%)
**Hypertension**	433(71.7%)
**Smoking**	145(24%)
**Systolic BP (mmHg)**	136(± 21)
**Diastolic BP (mmHg)**	78(± 11)
**Creatinine (μmol/l)**	184(± 71)
**eGFR (MDRD, ml/min/1.73 m^2^)**	36.8(± 13.9)
**Total cholesterol (mmol/l)**	4.8(± 1.0)
**Proteinuria (g/24 hours)**	0.3(0.1-0.9)
**Phosphate (mmol/l)**	1.12(± 0.26)
**Calcium (mmol/l)**	2.38(± 0.14)
**PTH (pmol/l)**	8.3(5.6-14.4)
**cFGF23 (RU/ml)**	116(67-203)
**Medication**	
**Active vitamin D (any)**	135(22.4)
**Phosphate binders (any)**	56(9.3)

Values are given as N (%), mean and standard deviations (± SD) for normally distributed data and medians and interquartile ranges (25%-75%) for skewed data.

CKD: chronic kidney disease; PKD: polycystic kidney disease; CVD: cardiovascular disease; BP: blood pressure; eGFR: estimated glomerular filtration rate; MDRD: four-point MDRD formula.

### Fibroblast growth factor 23 and classical cardiovascular risk factors (model 1)

**Figure 1:**
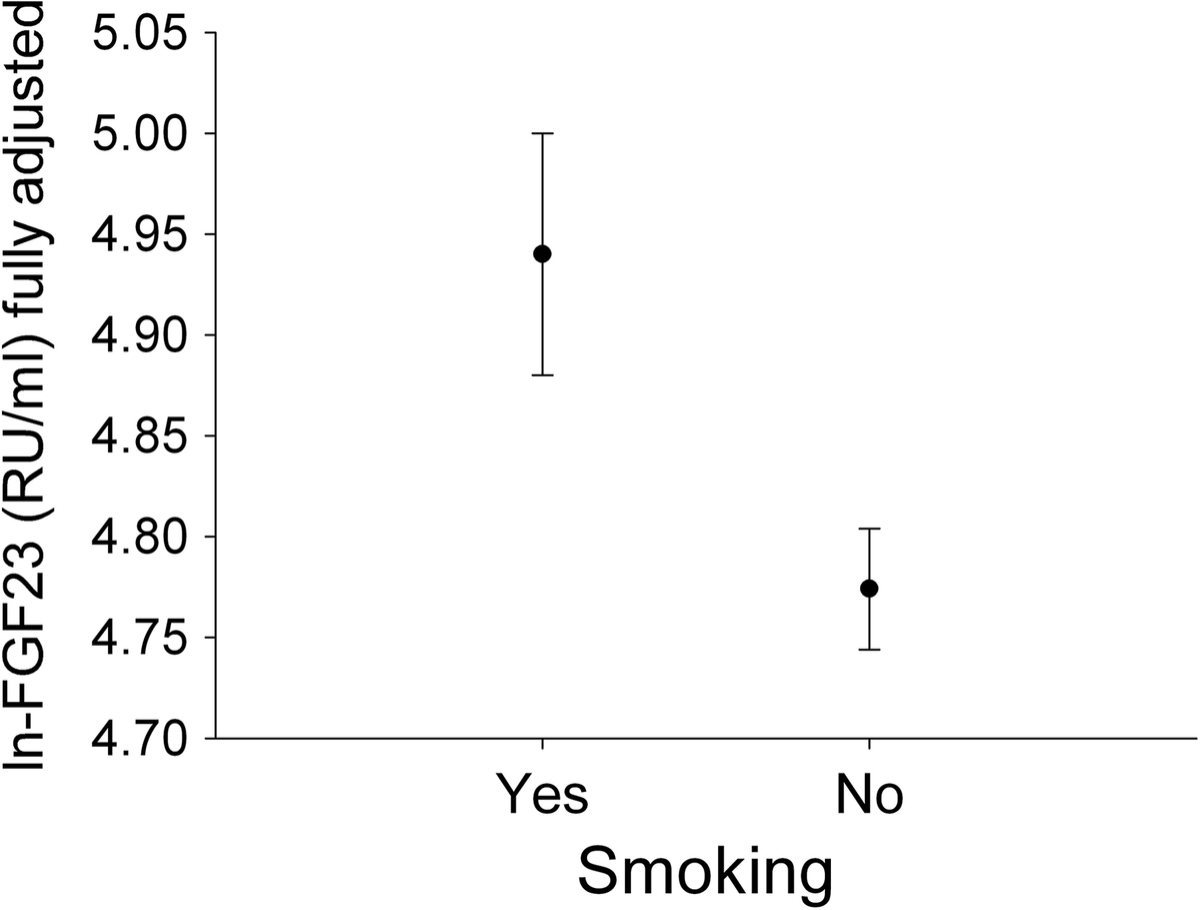
**Relation of smoking with FGF23**. FGF23 was log transformed and adjusted for eGFR, phosphate, natural logarithm of PTH, calcium, history of cardiovascular disease and history of diabetes mellitus. The error bars represent the standard error of the mean.

In multivariable regression analysis (Table 2), combining established cardiovascular risk factors, BMI (β = 0.105; p = 0.011), history of cardiovascular disease (β = 0.1; p = 0.018) and smoking (β = 0.136; p = 0.001) were positively associated with FGF23, while male sex was inversely associated with FGF23 (β = 0.89; p = 0.032). The relationship between smoking and FGF23 is shown in Figure 1.

**Table 2:** Model 1: FGF23 and traditional cardiovascular risk factors

Variable	B	Standard error	Beta	p-value
**Male**	-0.174	-0.081	-0.89	0.032
**Age (year)**	-0.003	-0.003	-0.041	0.354
**Systolic blood pressure (mmHg)**	0.002	0.002	0.043	0.308
**Smoking**	0.288	0.086	0.136	0.001
**BMI**	0.021	0.008	0.105	0.011
**Diabetes Mellitus**	0.154	0.089	0.073	0.084
**Total cholesterol (mmol/l)**	0.003	0.037	0.004	0.930
**History CVD**	0.198	0.084	0.100	0.018

Model 1: Relation of lnFGF23 with Framingham risk factors for cardiovascular disease after multivariable analysis. B reflects change in lnFGF23 per unit of the respective variable. For dichotomous variables (sex, smoking, diabetes, and history of CVD) B reflects lnFGF23 change in the presence of the depicted variable. Beta is the standardized B to compare influence on lnFGF23 between variables. BMI: Body mass index; CVD: cardiovascular disease

### Fibroblast growth factor 23 in the fully adjusted model (model 2)

**Figure 2:**
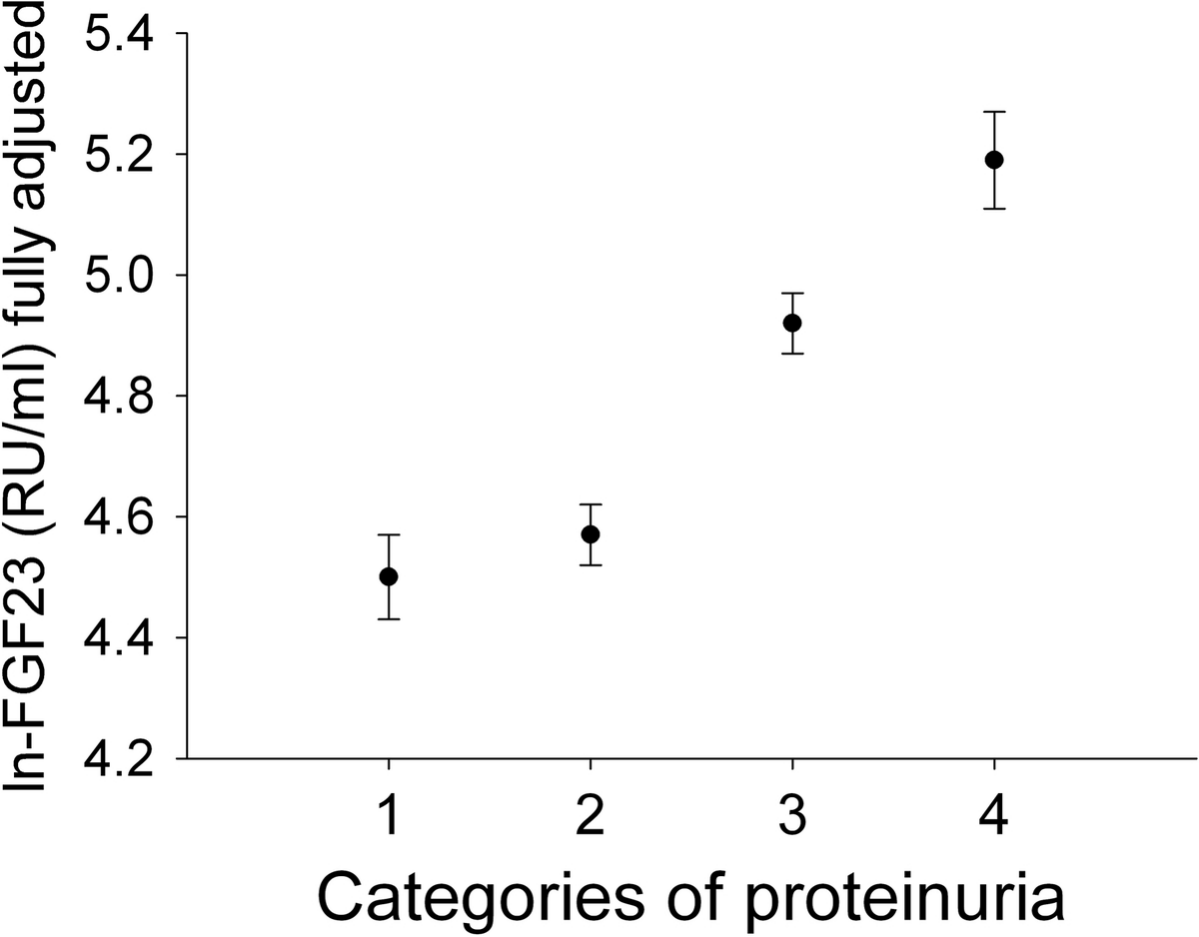
**Relation between proteinuria and FGF23**. FGF23 was log transformed and adjusted for eGFR, phosphate, natural logarithm of PTH, calcium, smoking, history of cardiovascular disease and history of diabetes mellitus. Categories of albuminuria: 1) no proteinuria; 2) proteinuria < 0.5 gram/24 hours; 3) proteinuria between 0.5 and 2.0 gram/24 hours and 4) proteinuria > 2 gram/24 hours. The error bars represent the standard error of the mean.

In this fully adjusted model, history of cardiovascular disease (β = 0.113; p = 0.002), presence of diabetes (β = 0.075; p = 0.035), smoking (β = 0.148; p < 0.001), phosphate level (β = 0.082; p = 0.024), PTH (β = 0.196; p < 0.001) and proteinuria (β = 0.113; p = 0.002) were positively associated with FGF23, while eGFR had an inverse association with FGF23 (β = -0.34; p < 0.001). After categorizing the level of proteinuria, a graded relationship between amount of proteinuria and adjusted level of FGF23 was revealed (Figure 2). All other factors had no significant association with FGF23 (Table 3).

**Table 3:** Model 2. Relation of lnFGF23 in the full adjusted model with cardiovascular and renal risk factors

Variable	B	Standard error	Beta	p-value
**eGFR (MDRD, ml/min/1.73 m^2^)**	-0.022	0.003	-034	< 0.001
**Phosphate (mmol/l)**	0.297	0.131	0.082	0.024
**lnPTH (pmol/l)**	0.244	0.047	0.196	< 0.001
**Calcium (mmol/l)**	0.393	0.220	0.062	0.075
**Proteinuria (gram/24 hours)**	0.064	0.021	0.113	0.002
**Male**	-0.094	0.071	-0.048	0.183
**Smoking**	0.313	0.073	0.148	< 0.001
**BMI**	0.003	0.007	0.017	0.638
**Diabetes Mellitus**	0.159	0.075	0.075	0.035
**History CVD**	0.224	0.071	0.113	0.002
**Age (year)**	0.001	0.003	0.006	0.868
**Systolic blood pressure (mmHg)**	-0.001	0.002	-0.02	0.586
**Total cholesterol (mmol/l)**	0.006	0.031	0.007	0.848
**Active vitamin D use**	0.096	0.079	0.044	0.223

Model 2: multivariable analyses of relation of lnFGF23 with Framingham risk factors (model 1) and renal risk factors. B reflects change in lnFGF23 per unit change in the variable. For dichotomous variables (sex, smoking, diabetes, and history of CVD) B reflects lnFGF23 change in the presence of the depicted variable. Beta is the standardized B to compare influence on lnFGF23 between variables.

eGFR: estimated glomerular filtration rate; MDRD: four-point MDRD-formula; BMI: Body mass index; CVD: cardiovascular disease

### Role of vitamin D status on the relationship between proteinuria and fibroblast growth factor 23

Since the relation between proteinuria, FGF23 and eGFR may be modulated by vitamin D status, levels of both 25vitD and 1,25vitD were determined in patients with proteinuria > 2 gr/24 hours (n = 63) and a random sample of equal size of those with proteinuria < 2 gr/24 hours. Levels of 25vitD were 51 (± 23) and 67 (± 24) nmol/l (non-significant) and levels of 1,25vitD were 44 (± 26) and 53 (± 25) pmol/l (p = 0.05) in patients with proteinuria > 2 gr/24 hours and patients with proteinuria < 2 gr/24 hours respectively. Details of these individuals are shown in Table 4.

**Table 4:** Baseline characteristics of subgroups with and without proteinuria > 2 gram/24 hours

Variable	Prot < 2 g/24 hrs	Prot > 2 g/24 hrs	p-value
**N**	63	63	
**eGFR (ml/min/1.73 m^2^)**	36.5 (± 14.1)	29.0(± 12.0)	0.002
**Phosphate (mmol/l)**	1.09(± 0.20)	1.19(± 0.24)	0.01
**Proteinuria (g/24 hrs)**	0.28[0.09-0.80]	3.30[2.60-5.50]	< 0.001*
**PTH (pmol/l)**	8(5-12)	11(7-20)	0.002
**cFGF23 (RU/ml)**	106 [65-186]	172 [100-242]	0.002
**25OD-D3 (nmol/l)**	67(± 25)	51(± 24)	0.001
**1,25(OH)_2-_D3 (pmol/l)**	53(± 25)	44(± 26)	0.05
**Active vitamin D use (%)**	21	37	0.05

Values are given as means (± SD) or medians [IQR].

*Per definition

In this subpopulation, levels of either 25D or 1,25D had no relation with FGF23 levels (p-values were 0.71 and 0.64 respectively). Phosphate level and smoking were positively associated, and eGFR negatively associated with FGF23 in this sub analysis. The magnitudes of the associations in this substudy were similar as in the entire cohort. In addition, the use of active vitamin D, as was assessed in the full data set and shown in Table 3, had no significant association with the level of FGF23.

## Discussion

Our study demonstrates that in patients with CKD FGF23 is significantly associated with smoking and proteinuria (Figures 1 &2). This association was strong and persisted after correcting for other well known cardiovascular and renal risk factors. In the fully adjusted model (Table 3) both smoking and proteinuria had a relatively high impact on FGF23 levels that was exceeded by eGFR and PTH only. Our data confirm the established relationship between FGF23 on the one hand and phosphate level, eGFR and PTH on the other [13]. This relationship possibly has a biological basis as FGF23 is physiologically regulated by phosphate levels [14], phosphate loading [15] and PTH [16].

Our observation of the association between smoking and FGF23 is novel. Possible explanations for this association are an influence of smoking on FGF23 production, an influence of smoking on FGF23 sensitivity or mere confounding by other factors that directly influence FGF23 levels. Smoking may increase FGF23 levels by affecting the metabolism of bone, the primary source of FGF23. Indeed, several studies have observed an association of smoking with osteoporosis [17,18]. However, there are no experimental data to support the assumption that smoking increases FGF23 production. Furthermore, if smoking directly increases FGF23 levels, we would have expected lower phosphate levels in smokers, which were not observed. Therefore, we consider it more likely that smoking reduces FGF23 sensitivity, thus necessitating an increased production to maintain phosphate excretion. The FGF23 receptor consists of heterodimerized FGFR1 and klotho, and is primarily located in the parathyroid and distal tubular cells in the kidney [19]. The expression of klotho is downregulated by oxidative stress [20], leading to dismantling of a functional FGF23-receptor. For this reason it is possible that smoking, *via *increased oxidant stress, decreases the number of functional FGF23 receptors in the kidney [21]. We cannot exclude the possibility that the association between FGF23 and smoking resulted from confounding by factors such as other life style differences between smokers and non-smokers. In contrast to others [22] we did not find a relation between body mass index and FGF23, in the full-adjusted model. The population studied by Marsell however was older, had better estimated GFR, and lower levels of FGF23. The putative relative contribution of BMI on FGF23 levels in the MASTERPLAN cohort may be overwhelmed by other factors, shown in Table 3. The relation we found between smoking and levels of FGF23 is in line with a recent study that addressed the predictive value of FGF23 on death and progression to end stage kidney disease [23]. In that study, the proportion of smokers increased in the higher quartiles of FGF23, however, the quantitative effect of smoking on FGF23 was not assessed. Correcting for several factors including smoking did not mitigate the strength of the association between FGF23 and the risk of death. However this does not imply that smoking cannot increase levels of FGF23. In another recent study in the general population, the percentage smokers did not differ between tertiles according to levels of FGF23 [24]. Whether higher FGF23 levels exist within individual tertiles among smokers, as compared with non-smokers, was not assessed.

The second major finding in our study is the graded relationship of FGF23 with proteinuria (Figure 2). This association can be explained in several ways. First it is possible that FGF23 has a direct effect on the glomerular filtration barrier, possibly through a direct effect on glomerular endothelial function [7] or glomerular hemodynamics. However, the evidence supporting this hypothesis is weak. Hypertension was not related to FGF23 and therefore cannot explain the rise in proteinuria with increasing FGF23 levels. Another explanation may be that proteinuria itself increases FGF23. This may be mediated by the association of proteinuria with vitamin D homeostasis [25]. Proteinuria however is associated with vitamin D deficiency and therefore a decline in FGF23 would have been expected, giving the observed increase in FGF23 levels seen upon supplementation with activated vitamin D in experimental models [26]. As patients with proteinuria received active vitamin D more frequently (shown in Table 4), this use of active vitamin D was added to the fully adjusted model (Table 3). Active vitamin D use however, did not explain the higher levels of FGF23 in patients with proteinuria. Another potential mechanism that could link proteinuria with FGF23 levels is secondary hyperparathyroidism, induced by deficiency of vitamin D that accompanies proteinuria. Adding lnPTH to the fully adjusted model however, did not mitigate the association of proteinuria with FGF23. A more likely explanation is that the toxic effects of proteinuria on tubular function [27,28] also disrupt local FGF23 signaling, leading to a compensatory increase in FGF23 production. A final explanation might be that both proteinuria and an increase in FGF23 are the consequence of the same underlying process, such as increased oxidative stress, that could lead to both proteinuria [29] and FGF23 resistance [20]. An association between proteinuria and FGF23 was also noted in the Heart and Soul study [6]. This study included patients with a mean GFR of 76 ml/min, and only 4% had macroalbuminuria. Our study, with mean GFR of 37 ml/min/1.73 m2 and over 10% of patients having proteinuria > 2 g/day, extends these observations to patients with more severe CKD and higher levels of proteinuria. Fliser and co-workers found an association between FGF23 and progression of CKD, even after correction for albumin-to-creatinine ratio (ACR), but that correction attenuated the predictive value of FGF23 [30]. In that study the precise relation between FGF23 and proteinuria was not studied. In the study by Isakova the association between FGF23 and progression to end stage kidney disease was no longer significant after correcting for albumin-to-creatinine ratio, eGFR, albumin and hemoglobin, but the risk for mortality remained significant [23].

We found no modulating effect of either 25D or 1,25D levels on FGF23. This lack of evidence for an association of FGF23 with levels of vitamin D metabolites can be explained by the fact that active vitamin D induces FGF23 production [26] while FGF23 in turn catabolizes 25D and 1,25D [31], thus counterbalancing possible relationships between vitamin D and FGF23.

It needs to be pointed out that some associations present in Model 2 were no longer present in the analysis in which vitamin D was included. There are two factors to explain this. Firstly the number of participants in this latter analysis is much lower (604 in model 2; 126 in vitamin D analysis). In addition proteinuria was dichotomized in two categories (more or less than 2 gr/24 hours), which reduces the discriminatory value of this variable in linear regression.

Accumulating evidence points to FGF23 as a novel risk factor for mortality and progression of CKD [3,23]. Our findings show that a part of the increased cardiovascular risk of smoking [32] and proteinuria [33] in CKD may thus be explained by its effects on FGF23, assuming that FGF23 has direct detrimental effects. This may indicate that FGF23 has a causal role in cardiovascular complications in CKD, but this still is still debated, because of the possibility of residual confounding within these studies and the lack of definitive proof of a pathobiological mechanism by which FGF23 presumably induces harm. Recent evidence however does suggest that FGF23 is directly involved in the evolution of pathological left ventricular hypertrophy in mice [9]. For some factors, like GFR[1???] and phosphate level [34,35], both reasonably established novel risk factors for cardiovascular disease, its relationship with FGF23 is well described. However, previous studies demonstrated that the predictive value of FGF23 for clinical endpoints remained significant, even after correcting for these factors that are involved in its production, providing additional arguments for the notion that FGF23 might be more than just a risk marker.

Some limitations of our study need to be mentioned. First, our study is a cross-sectional observational analysis, thus we cannot determine temporal relationships and causality. Next, it could be possible that FGF23 influences proteinuria, instead of the other way around. Indirect evidence for the latter comes from the observation that protein (and phosphate) restriction improves albuminuria [36]. In addition, we also cannot answer the clinically relevant question whether modulating proteinuria or smoking cessation can lower FGF23 and if FGF23 decline accomplished by these measures reduces cardiovascular risk (independent from risk reduction by smoking cessation alone and proteinuria reduction). Such a question requires a RCT.

There are currently two techniques available to measure FGF23: intact FGF23 and c-terminal FGF23. The former assay is positive only in the presence of full-length FGF23, while the latter also measures the c-terminal truncated FGF23. In our study we did not measure intact FGF23, but in advanced CKD the two assays have been shown to perform similarly [37]. We were able to test 25D and 1,25D levels in a subset only, which weakens our conclusion that vitamin D metabolites had no relation with FGF23. Indeed, a recent study suggests that higher FGF23 was associated with lower 1,25D levels [4]. However, proteinuria was not measured in that study.

Strengths of our study are the large sample size, the prospective nature of data collection and the use of 24 hour urine collections instead of using albumin-to-creatinine ratio in spot urine samples, which provides more reliable quantitative data [38].

Our findings can have important implications. If FGF23 indeed has a causative role in cardiovascular disease and is involved in progression of CKD, an important next step would be to test the hypothesis that dietary or pharmacological interventions aimed at a reduction of FGF23 improves these outcomes. The obvious tools to lower FGF23 would be a phosphate-restricted diet [15], phosphate-binder therapy [39-41] or calcimimetics [42]. Our findings support the hypothesis that early intervention aiming at reducing proteinuria and cessation of smoking may be another tool to reduce FGF23.

## Conclusion

In patients with CKD FGF23 is significantly associated with proteinuria and smoking, along with other, already established associations, like eGFR (inverse relation), plasma phosphate level, PTH, history of cardiovascular disease and diabetes mellitus. The clinical relevance of this finding is the fact that both proteinuria and smoking are modifiable. Future prospective randomized trial, or post-hoc analyses of existing cohorts, can reveal if targeted anti-proteinuric therapy lowers FGF23, and if this assumed reduction is independently associated with improved outcome. Since the causal relationship between FGF23 and proteinuria can be two-sided, an additional question is the impact of targeting FGF23 to lower proteinuria. The relation of FGF23 with smoking provides an additional argument to discourage this habit.

## Competing interests

JW received lecture fees and travel reimbursements by Amgen and Genzyme. AZ received and lecture fees and travel reimbursements by Genzyme. PB received travel reimbursements by Amgen. PW received lecture fees from Amgen and research grant from Genzyme. The other authors reported no disclosures/conflicts of interest.

## Authors' contributions

MV analyzed the data, performed FGF23 measurements, and wrote the manuscript. AZ analyzed the data, performed statistical analysis, managed the database, and contributed substantially to the writing of the manuscript. AH performed analyses of FGF23, vitamin d metabolites and analyzed the data. MB analyzed the data, contributed to the statistical analysis and its interpretation, PW participated in the analysis of data and writing of the manuscript. PB designed the trial, supervised its conduct, analyzed the data and contributed to the manuscript. JW designed the trial, supervised its conduct, analyzed the data and contributed to the manuscript. All authors read and approved the final manuscript.

## Pre-publication history

The pre-publication history for this paper can be accessed here:

http://www.biomedcentral.com/1471-2369/13/20/prepub
